# What Is the Optimal Length of Stay for Effective Inpatient Neurorehabilitation? A Retrospective Cohort Study

**DOI:** 10.1002/hsr2.71502

**Published:** 2025-11-11

**Authors:** Elham Loni, Mohammad Sayadnasiri, Nazila Akbarfahimi, Mehdi Basakha, Razieh Bidhendi‐Yarandi

**Affiliations:** ^1^ Clinical Research Development Center of Rofeideh Rehabilitation Hospital University of Social Welfare and Rehabilitation Sciences Tehran Iran; ^2^ Department of Clinical sciences University of Social Welfare and Rehabilitation Sciences Tehran Iran; ^3^ Department of Psychiatry University of Social Welfare and Rehabilitation Sciences Tehran Iran; ^4^ Department of Occupational Therapy University of Social Welfare and Rehabilitation Sciences Tehran Iran; ^5^ Social Determinants of Health Research Center, Social Health Research Institute University of Social Welfare and Rehabilitation Sciences Tehran Iran; ^6^ Department of Social Welfare Management, School of Social Health University of Social Welfare and Rehabilitation Sciences Tehran Iran; ^7^ Department of Biostatistics and Epidemiology, School of Social Health University of Social Welfare and Rehabilitation Sciences Tehran Iran; ^8^ Neuromusculoskeletal Rehabilitation Research Center University of Social Welfare and Rehabilitation Sciences Tehran Iran

**Keywords:** inpatient rehabilitation, length of stay, multiple sclerosis, spinal cord injury, stroke

## Abstract

**Background and Aims:**

The length of stay (LOS) in inpatient rehabilitation varies significantly and remains ambiguous. Therefore, we sought to determine the optimal length of inpatient rehabilitation.

**Methods:**

This retrospective cohort study was conducted at a specialized inpatient rehabilitation hospital. Clinical data from 258 patients with neurological disorders, including stroke, spinal cord injury, and multiple sclerosis, were collected and analyzed. The primary outcome measure was the change in patients' functional status as assessed by the Functional Independence Measure (FIM). FIM scores were recorded at 21‐day intervals throughout the hospitalization period. To evaluate the effectiveness of inpatient rehabilitation over time, trends in FIM score changes were examined using effect size calculations. Additionally, Generalized Estimating Equations (GEE) were employed to analyze repeated measures and account for intra‐subject correlations across the different time points.

**Result:**

In spinal cord injury (SCI) survivors, the trend of improvement continued with significant effectiveness:1.847 (95% CI: 1.445, 2.249), 3.320 (95% CI: 2.495, 4.144) and 3.036 (95% CI: 1.426, 4.645) during the first, second and discharge assessments, respectively, compared to Admission time. Patients with multiple sclerosis (MS) shown significant effectiveness only during the first 21‐day period: 1.276 (95% CI: 0.723, 1.830). Stroke survivors also showed significantly improved their FIM over the three 21‐day periods: first period:1.637 (95% CI: 1.284, 1.990), second period: 2.952 (95% CI: 2.106, 3.802), and discharge:3.804 (95% CI: 2.944, 4.668).

**Conclusions:**

LOS exceeding 42 days resulted in a significant increase in the FIM scores of stroke patients. However, an extended stay of 21 days in the MS group and 42 days in the SCI group did not demonstrate significant improvement.

## 1 Introduction

Neurorehabilitation is a significant area of rehabilitation aimed at providing services to individuals suffering from neurological diseases or disabilities; such as stroke, multiple sclerosis, spinal cord injuries and brain injuries. These services can alleviate considerable emotional, physical, and financial burden on patients and their families, ultimately improving their quality of life. These services are delivered through a variety of methods in both outpatient and inpatient settings [1, 2, 3] Neurological diseases have a wide range of effects. Despite advancements in medication, intervention, and surgery for acute treatment over the years, due to the nature of neurological damage, some disabilities persist in certain individuals following the onset of the disease, even after acute phase treatment. These individuals require rehabilitation services, primarily delivered through team‐based approaches. For instance, two‐thirds of stroke patients eventually face disabilities after the acute phase of the illness and evidence suggests that early, intensive rehabilitation with clear objectives, predominantly as multidisciplinary, can lead to better outcomes [3, 4, 5].

Individuals are candidates for this level of rehabilitation when their vital signs are stable and they can receive services for at least 3 hours a day and 5 days a week. These services are primarily provided in rehabilitation centers separate from acute care settings. During this hospitalization, the duration of which depends on the policies of each region and patients may stay for several days to weeks. The discharge time is contingent upon the rehabilitation team's assessment and is established with a specific plan [6, 7] The Functional Independence Measure (FIM) is a standardized metric for assessing independence in self‐care, widely used in various studies to demonstrate individuals' functional gains during rehabilitation in some healthcare systems and insurance reimbursement for rehabilitation is conditional on evidence of continuous improvement in the FIM score [8, 9] there are different factors that have influence on FIM change during rehabilitation such as time of start of rehabilitation after injury, FIM score at admission, number of rehabilitation sessions and length of hospital stay [10] The length of stay is a significant factor in evaluating health and safety systems for controlling costs, managing hospital complications, and resource management. It can also influence the effectiveness of inpatient rehabilitation outcomes [11, 12] Previous studies show considerable variation in rehabilitation stay duration across different countries and neurological conditions, ranging from 21 to 147 days [13, 14] What remains unclear is the effective length of stay of inpatient rehabilitation. Despite various reports on inpatient rehabilitation outcomes, there is still no clear consensus on the optimal length of stay across neurological conditions. Additionally, few studies have investigated how functional outcomes evolve over time in distinct neurological populations, using comparable metrics like FIM. This study aims to analyze time‐dependent changes in functional independence during inpatient rehabilitation across patients with stroke, SCI, and MS. By identifying the trajectory and timing of maximal functional improvements, this study seeks to address a significant gap in the literature and provide data to inform care planning, optimize LOS, and guide policy decisions for efficient rehabilitation service delivery.

## 2 Methods

The present study is a retrospective cohort analysis utilizing clinical data from patients hospitalized at Rofeideh Rehabilitation Hospital. This facility is currently the only specialized inpatient neurorehabilitation hospital in the country providing standardized multidisciplinary care; therefore, all data has necessarily been collected from this center. The study was conducted after obtaining approval from the ethics committee and receiving an ethics code from the University of Social Welfare and Rehabilitation Sciences with the ethical code of IR. USWR. REC.1402.093. It reviewed all available patient documents through a census method within the timeframe of August 2021 to August 2022, focusing on patients with neurological disorders (spinal cord injury, stroke, multiple sclerosis, Traumatic brain injury) who received individualized multidisciplinary rehabilitation (physiotherapy, occupational therapy, speech therapy) based on diagnosis and clinical status, mostly in subacute to early chronic phase.

### 2.1 Exclusion Criteria

- Patients who were hospitalized solely due to pressure ulcers and did not receive standard rehabilitation services.
- Patients who developed a wound during their hospitalization that significantly disrupted the receipt of standard rehabilitation services.
- Patients who had only one FIM assessment (Probably due to early discharge).

### 2.2 Measurement Tools

- Demographic Information: This checklist was developed by researchers and includes questions regarding the demographic characteristics of the samples and the background variables of the rehabilitation seekers, such as: age, sex, type of illness, duration of hospitalization (in days), and number of rehabilitation services (count).
- Functional Independence Measure (FIM): This is a common tool in rehabilitation sciences created by Granger and colleagues to assess independence or dependence when performing daily functional activities. This tool has been validated for various groups with neurological disorders. The scale consists of 18 items organized into six subgroups to assess motor activities (13 items: self‐care, sphincter control, mobility, and movement) and cognitive activities (five items: communication and social cognition). Scoring is done on a seven‐point scale, where a score of one indicates complete assistance for activities, and a score of seven indicates full independence in performing these activities. The maximum score on this scale is 126, and the minimum is 18, with the administration of this test taking approximately 45 min based on the abilities of the participant. This tool was examined in Iran by Naghdi and colleagues (2015), achieving a Cronbach's alpha ranging from 0.75 to 0.96 and an interclass correlation coefficient ranging from 0.76 to 0.98 [15] In our study, this measure was assessed and recorded every 21 days by occupational therapist.

### 2.3 Data Analysis

To describe and analyze the data, descriptive and inferential statistics will be employed. Effect sizes before and after treatment have been estimated. Given the nature of the data, paired t‐tests, analysis of covariance, and generalized estimating equations (GEE) will be utilized for the analysis of repeated measures (1‐admission time, 2‐first 21‐day period, 3‐second 21‐day period (42 days), and 4‐discharge). The trend of changes in mean scores over four measurements has been investigated. It is noteworthy that the average score of functional independence was calculated by dividing the raw score of functional independence by the rehabilitation session rate (number of sessions over the patient's length of stay). The analysis was conducted using STATA‐17 software.

## 3 Results

### 3.1 Descriptive Findings

After reviewing the exclusion criteria data from 258 cases (including 36 multiple sclerosis, 85 stroke, and 137 spinal cord injury cases) were included in the final analysis. Due to incomplete FIM data (Incomplete FIM data refers to cases where fewer than two FIM assessments were available, which precluded measurement of functional change), no traumatic brain injury cases were included in the study.

Among 258 patients with mean age 46.3 (±17), 53% was admitted in spinal cord injury ward. The mean length of stay was 41.5 days (±17.9) among all patients. Analysis of the length of stay indicates that patients with the spinal cord injury had a longer stay about 46.6 days (±17.9) compared to the other. There were differences in the amount services received among the various diagnosis. Each patient in the spinal cord injury ward received an average of 6.6 (±1.5) rehabilitation services (including physiotherapy, occupational therapy, or speech therapy). In contrast, multiple sclerosis patients had the lowest service utilization, averaging 4.4 services per day ( ± 1.1). more details can be found in Table 1.

**Table 1:** Demographic characteristic, length of stay and number of rehabilitation services.

Diagnosis	Mean age	N/percentage	Sex	Male	Mean length of stay (days)	Mean rehabilitation services (*N*)
				Female		
Spinal cord injury	34.2 ( ± 16)	137 (53.1%)	79 (57.6%)		46.6 ± 17.9	6.6 ± 1.5
			58 (42.4%)			
stroke	61.9 ( ± 13)	85 (32.9%)	63 (74%)		35.6 ± 14.7	5.8 ± 0.9
			22 (26%)			
Multiple sclerosis	35.2 ( ± 12)	36 (14%)	14 (39%)		36.6 ± 19.8	4.4 ± 1.1
			22 (61%)			
Total	46.3 ( ± 17)	258 (100%)	156 (60.5%)		41.5 ( ± 17.9)	6 ± 1.5
			102 (39.5%)			

### 3.2 Effectiveness of Neurorehabilitation Services

It is observed that mean of patients' FIM at the admission time was 62.1 ( ± 20.4) totally. The mean FIM for stroke was the lowest (54.2 ( ± 24)), while patients with multiple sclerosis had the highest mean FIM (71.6 ( ± 22.6)) at admission. The status of patients' FIM has been reassessed at 21 days' intervals. The mean FIM score for all hospitalized patients upon discharge was 78.5 ( ± 21.7), indicating an improvement of 16 points. Patients in the stroke unit have shown the most significant improvement during their hospital stay, with an increase of 22.3 points ( ± 26.1 points). In contrast, the FIM score of patients in the MS ward upon discharge was slightly lower than at the time of admission surprisingly. The overall FIM score for patients during the first, second, and third 21‐day periods indicated increases of 15%, 9%, and 0.1%, respectively, compared to the preceding period (Table 2).

**Table 2:** Functional independence measure score (FIM) in all groups and periods.

Diagnosis	FIM score	Mean (SD)	FIM change (percentage)^a^	Minimum	maximum
SCI	Admission time	64.5 (15.1)		22	11
	21 days^b^	75.79 (17.9)	17%	22	119
	42 days^c^	83.1 (18.8)	10%	23	120
	Discharge	84 (18.6)	1%	24	113
Stroke	Admission time	54.2 (24)		12	111
	21 days	63.4 (24.4)	17%	13	112
	42 days	71.2 (28.2)	12%	17	117
	Discharge	73 (26.1)	1%	23	114
MS	Admission time	71.6 (22.6)		40	124
	21 days	76 (23.2)	6%	44	124
	42 days	73 (20.9)	−4%	44	119
	Discharge	70.3 (21.9)	−4%	41	117
Total	Admission time	62.1 (20.4)		12	124
	21 days	71.7 (21.7)	15%	13	124
	42 days	78.6 (22.6)	9%	17	120
	Discharge	78.5 (21.7)	0.1%	20	117

^a^ FIM change: FIM score changes compared to the preceding period.

^b^ First 21‐day period.

^c^ Second 21‐day period.

**Figure 1:**
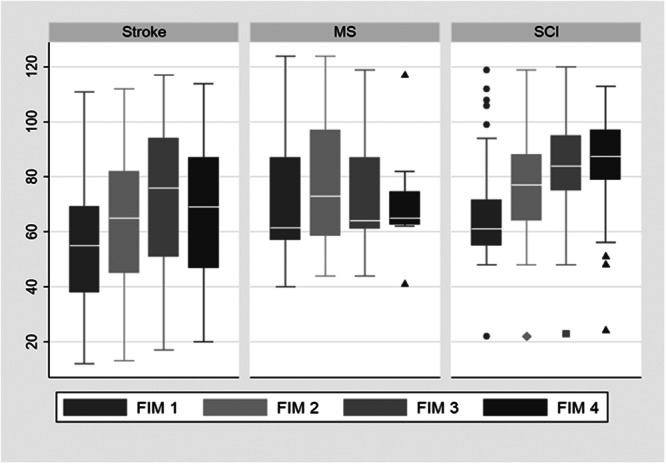
Periodic assessment of FIM score based on diagnosis.

The trend of FIM scores for patients in various diagnosis of Rofeideh Hospital is also illustrated in Figure 1.

**Figure 2:**
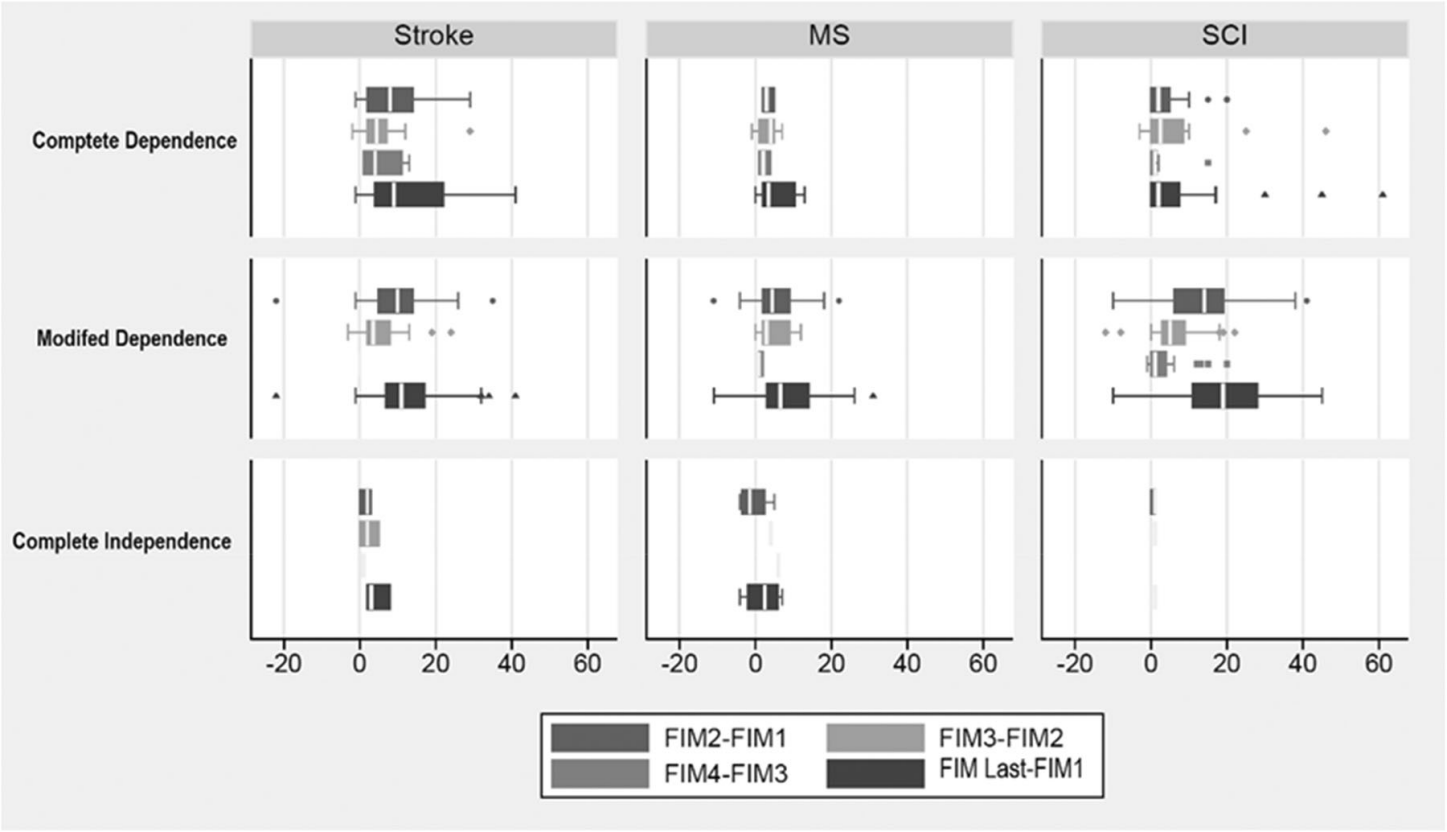
Change of FIM score during hospitalization considering initial condition.

The improvement observed during the first 21‐day period among all patients with varying initial physical conditions was greater than in the second and third periods. However, there is one exception. Patients admitted to the spinal cord injury unit who had complete functional independence benefitted more from services during the second 21‐day period than in the first. (Patients' status is classified not only based on their hospital stay but also according to their initial FIM score, with changes in each category presented independently. For classifying the initial status of patients, a FIM score of 108 or higher is considered complete independence, a score between 37 and 107 indicates relative independence, and a score below 37 is regarded as complete dependence) [16]. It can be inferred from Figure 2 that stroke patients, regardless of their initial condition, experienced the greatest improvement from rehabilitation services. Additionally, as the length of the hospital stay increased, the improvement in FIM change decreased for all patient groups.

**Figure 3:**
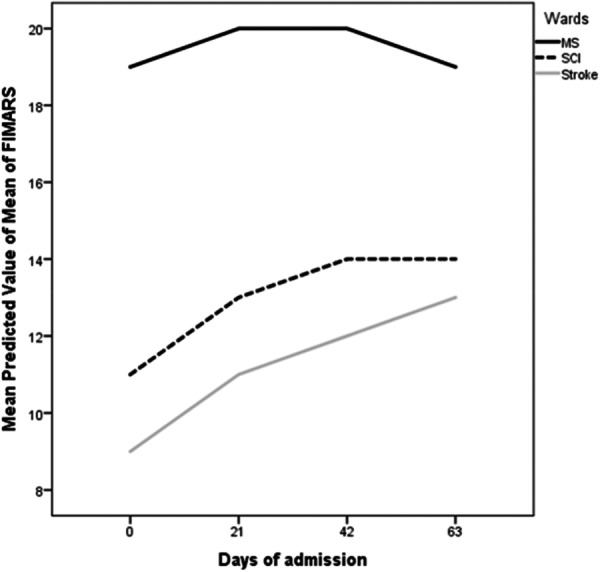
Periodic assessment of FIM score based on diagnosis.

Table 3 and Figure 3 illustrates the results of GEE modeling for the variable of ratio of FIM score to Average Rehabilitation Sessions (FIMARS) to consider the trend of changes in FIM scores adjusted by the rate of rehabilitation during the hospitalization of patients.

**Table 3:** the results of GEE modeling for the variable of ratio of FIM score to average rehabilitation sessions (FIMARS).

Diagnosis	Main effects time^a^	Beta (95% CI)	*p* value
**SCI**	The second FIMARS compared to the admission FIMARS	1.847 (1.445, 2.249)	0.001*
	The third FIMARS compared to the admission FIMARS	3.320 (2.495, 4.144)	0.001*
	The fourth FIMARS compared to the admission FIMARS	3.036 (1.426, 4.645)	0.001*
	The third FIMARS compared to the second FIMARS	1.473 (1.222, 1.725)	0.001*
	The fourth FIMARS compared to the third FIMARS	−1.073 (−3.387, 1.240)	0.363
**MS**	The second FIMARS compared to the admission FIMARS	1.276 (0.723, 1.830)	0.001*
	The third FIMARS compared to the admission FIMARS	1.273 (−0.297, 2.843)	0.112
	The fourth FIMARS compared to the admission FIMARS	0.199 (−2.408, 2.807)	0.881
	The third FIMARS compared to the second FIMARS	−0.004 (−1.373, 1.380)	0.996
	The fourth FIMARS compared to the third FIMARS	−0.284 (−2.155, 1.587)	0.766
**Stroke**	The second FIMARS compared to the admission FIMARS	1.637 (1.284, 1.990)	0.001*
	The third FIMARS compared to the admission FIMARS	2.952 (2.106, 3.802)	0.001*
	The fourth FIMARS compared to the admission FIMARS	3.804 (2.944, 4.668)	0.005*
	The third FIMARS compared to the second FIMARS	1.316 (0.952, 1.680)	0.012*
	The fourth FIMARS compared to the third FIMARS	0.85 (0.060, 1.640)	0.035*

^a^ Second: 21‐day period, third: 42‐day period, fourth: discharge.

* Significant level < 0.05.

Results indicated that, rehabilitation services significantly increased the trend of functional independence scores over time in patients with spinal cord injuries (SCI). During the first (21 days), second (42 days) and discharge periods, the trend of functional independence scores continued to increase with significant effectiveness estimated Beta: 1.847 (95% CI: 1.445, 2.249), 3.320 (95% CI: 2.495, 4.144) and 3.036 (95% CI: 1.426, 4.645), compared to the admission, respectively. However, the rate of increase has decreased from the second period onwards in a way that the estimated coefficients were 1.473 (95% CI: 1.222, 1.725) for the third to the second assessment and −1.073 (95% CI: −3.387, 1.240) for the fourth to the third assessment, in which from the 42 days until discharge period no significant effectiveness was detected.

Regarding MS survivors, rehabilitation services have shown a statistically significant positive effect during the first 21‐day period. Specifically, the average score for FIM in these patients increased by 1.276 (95% CI: 0.723, 1.830) during 21‐day period compare to the admission. However, the changes in functional independence scores during the third 21‐day period until discharge were not statistically significant.

In patients who have experienced a stroke, rehabilitation services have significantly improved their FIM over the three 21‐day periods. The results indicate that from the second period to discharge time the average FIM score increased significantly compared to the admission time by 1.637 (95% CI: 1.284, 1.990), 2.952 (95% CI: 2.106, 3.802) and 3.804 (95% CI: 2.944, 4.668), respectively. However, the results indicate that from the second hospitalization period onward, the rate of increase in functional independence scores has diminished by 1.316 (95% CI: 0.952, 1.680) and 0.85 (95% CI: 0.060, 1.640) (Table 3, Figure 3).

## 4 Discussion

The care provided to patients in the inpatient rehabilitation hospital is multidisciplinary rehabilitation. The effectiveness of simultaneously delivering various specialized rehabilitation services to patients has been emphasized in several studies [17, 18]. In this study the mean FIM score of patients at admission was 62.1. By the time of discharge, the average FIM score increased to 78.5, indicating an improvement of 16 points. This finding is consistent with the results of Turner Stokes and colleagues [7] and demonstrates the effectiveness of inpatient rehabilitation. However, what raises a question for us is whether a longer duration of hospitalization lead to higher Functional Independence Measure score?

In this study, the mean length of stay for patients was 41.5 days, with the longest stay being 46.6 days for patients with spinal cord injuries. The variation in rehabilitation length of stay across different studies and countries ranges from 116 days in Japan to 47 days in Russia for spinal injuries, indicating that a standard duration for rehabilitation stays has yet to be established. This duration can certainly be influenced by factors such as the severity of the injury and the baseline level of function [14, 19, 20]. Onur Altunas and collogues, have shown that improvement in patients with stroke is influenced by duration of services provided to them and They believed that rehabilitation should continue to the extent that functional independence improves, rather than assuming that a longer rehabilitation period necessarily leads to greater improvement in functional independence [10]. However, the results of the present study indicate that the total FIM score of hospitalized patients increased by 15%, 9%, and 0.1% during the first, second, and third 21‐day periods, respectively, compared to the prior period.

By using GEE analysis, the individual trends in the changes in functional independence scores were examined. The relationship between the changes in FIM scores and the average number of rehabilitation sessions was analyzed to minimize the confounding effect of varying amount of rehabilitation services among different patients.

In patients with spinal cord injuries, functional independence significantly improved during the first three assessment periods. The increases in functional independence measure (FIM) scores at 21 days, 42 days, and discharge were substantial and statistically significant, underscoring the benefits of early rehabilitation. The nonsignificant difference in scores between the third and fourth assessment periods suggests that although patients continue to make progress, the gains taper off as the length of stay increases. Our results were consistent with studies indicating that the first 6–8 weeks of intervention for SCI patients is the efficient time [21]

Among patients suffering from MS, significant gains in FIM scores were only during the first 21‐day period. This early progress may reflect responsiveness to structured rehabilitation during the acute or subacute phase. However, the absence of significant gains in the later phases suggests a limited cumulative effect of continued inpatient rehabilitation for this group. This could be due to the progressive nature of MS, fluctuations in symptom severity, and potential fatigue or cognitive challenges that hinder longer‐term functional improvements [22]. These findings emphasize the importance of individualized rehabilitation planning and possibly integrating outpatient or home‐based programs after initial inpatient gains.

Patients with stroke showed consistent and statistically significant improvements in FIM scores across all periods, highlighting the effectiveness of rehabilitation in this population. Nevertheless, similar to SCI patients, the rate of improvement declined over time. This deceleration suggests that although patients continue to gain function, the marginal returns of continued inpatient rehabilitation decrease. These results could be align with well‐established stroke recovery models, indicating that most functional recovery occurs within the first 3 months' post‐stroke, and the rate of improvement slows as neuroplastic adaptation plateaus [23]

These findings highlight the critical importance of early and intensive rehabilitation across neurological populations. While the gains from continued inpatient therapy diminish over time, the initial improvements are substantial and clinically meaningful. For patients with conditions such as MS, where gains plateau earlier, it may be beneficial to explore alternative or adjunctive rehabilitation strategies, including cognitive and vocational therapy, tailored to disease‐specific challenges. They also point to the need for more personalized rehab plans that evolve with each patient's condition and stage of recovery.

According to this model, in spinal cord injury the improvement trends showed no significant increases after 42 days, and for multiple sclerosis patients, no increases were observed after 21 days (Figure 3, Table 3). Numerous studies have examined the efficacy of inpatient rehabilitation for multiple sclerosis patients; however, most of these studies report short hospitalization periods (3 to 4 weeks) has been deemed effect [13, 24], aligning with our findings in terms of timing. Based on our study, stroke patients experienced the highest improvement during hospitalization, with a mean increase of 22.3 units ( ± 26.1). According to GEE modeling, the third phase of rehabilitation (beyond 42 days) significantly improved functional independence only for stroke patients. Two studies have also demonstrated that extended inpatient rehabilitation services had a notable impact on improving these patients' FIM [25, 26]. Furthermore, according to classify patients' initial statuses, it can be inferred from Figure 2 that stroke patients, regardless of their initial condition, achieved the most significant improvement from rehabilitation services.

### 4.1 Strengths and Limitations

A major strength of this study is the stratified GEE analysis across three distinct neurological conditions, allowing for comparative insights into how different pathologies respond to structured rehabilitation. However, the study is limited by the observational nature of the data, which may be influenced by unmeasured confounding variables such as baseline comorbidities, psychosocial support, or rehabilitation intensity. Furthermore, long‐term outcomes post‐discharge were not assessed, limiting the understanding of sustained functional independence. A lack of sufficient information about patient details, such as the duration of injury and the level and severity of damage and potential impact of complications, is another limitation of our study, which could serve as a basis for future research and more accurate result estimation. The availability of more diverse tools for measuring effectiveness could enhance the precision of the study, as we only utilized the FIM tool in this study.

## 5 Conclusion

The changes observed in FIM score of patients at discharge were evident in all patients; however, these changes resulting from various rehabilitation periods showed significant differences among patients based on diagnoses. It was found that a hospitalization period exceeding 42 days resulted in a significant improvement in the functional independence of stroke patients, based on the modeling of changes in independence scores. In patients with spinal cord injuries, hospitalization beyond 42 days did not result in a notable change compared to their previous status, and for MS survivors, an extended stay of 21 days also did not show significant improvement in their condition. These findings can directly guide clinicians and policymakers in designing time‐sensitive, diagnosis‐specific rehabilitation plans to improve resource allocation, patient outcomes, and cost‐efficiency.

## Author Contributions

**Elham Loni:** conceptualization, data curation, visualization, writing – original draft, methodology, investigation, supervision, writing – review and editing. **Mohammad Sayadnasiri:** conceptualization, data curation, methodology, investigation. **Nazila Akbarfahimi:** writing – review and editing, data curation, conceptualization, methodology, investigation, supervision. **Mehdi Basakha:** conceptualization, data curation, formal analysis, visualization, writing – original draft, writing – review and editing, supervision, investigation, methodology, validation, funding acquisition. **Razieh Bidhendi‐Yarandi:** investigation, methodology, validation, formal analysis, writing – original draft.

## Ethics Statement

The present study protocol was reviewed and approved by the Research Ethics Committee of the University of Social Welfare and Rehabilitation Sciences (approval code: IR. USWR. REC.1402.093). As the research involved retrospective analysis of anonymized hospital records with no direct patient contact and no access to personal identifiers, the committee granted a waiver of informed consent. All data were handled in accordance with institutional policies and the principles of the Declaration of Helsinki to ensure confidentiality and privacy.

## Conflicts of Interest

The authors declare no conflicts of interest.

## Transparency Statement

Mehdi Basakha affirms that this manuscript is an honest, accurate, and transparent account of the study being reported; that no important aspects of the study have been omitted; and that any discrepancies from the study as planned (and, if relevant, registered) have been explained.

## Data Availability

The data that support the findings of this study are available on request from the corresponding author. The data are not publicly available due to privacy or ethical restrictions. The authors confirm that the data supporting the findings of this study are available within the article. Due to privacy and ethical restrictions, raw patient data are not publicly available but may be shared upon reasonable request and with permission from the ethics committee.
